# Phylometrics: a pipeline for inferring phylogenetic trees from a sequence relationship network perspective

**DOI:** 10.1186/1471-2105-11-S6-S18

**Published:** 2010-10-07

**Authors:** Samuel A Smits, Cleber C Ouverney

**Affiliations:** 1 Department of Biological Sciences, San José State University, One Washington Square, San José, California, 95192-0100, USA

## Abstract

**Background:**

Comparative sequence analysis of the 16S rRNA gene is frequently used to characterize the microbial diversity of environmental samples. However, sequence similarities do not always imply functional or evolutionary relatedness due to many factors, including unequal rates of change and convergence. Thus, relying on top BLASTN hits for phylogenetic studies may misrepresent the diversity of these constituents. Furthermore, attempts to circumvent this issue by including a large number of BLASTN hits per sequence in one tree to explore their relatedness presents other problems. For instance, the multiple sequence alignment will be poor and computationally costly if not relying on manual alignment, and it may be difficult to derive meaningful relationships from the resulting tree. Analyzing sequence relationship networks within collective BLASTN results, however, reveal sequences that are closely related despite low rank.

**Results:**

We have developed a web application, Phylometrics, that relies on networks of collective BLASTN results (rather than single BLASTN hits) to facilitate the process of building phylogenetic trees in an automated, high-throughput fashion while offering novel tools to find sequences that are of significant phylogenetic interest with minimal human involvement. The application, which can be installed locally in a laboratory or hosted remotely, utilizes a simple wizard-style format to guide the user through the pipeline without necessitating a background in programming. Furthermore, Phylometrics implements an independent job queuing system that enables users to continue to use the system while jobs are run with little or no degradation in performance.

**Conclusions:**

Phylometrics provides a novel data mining method to screen supplied DNA sequences and to identify sequences that are of significant phylogenetic interest using powerful analytical tools. Sequences that are identified as being similar to a number of supplied sequences may provide key insights into their functional or evolutionary relatedness. Users require the same basic computer skills as for navigating most internet applications.

## Background

The breadth of knowledge of microbial diversity continues to rapidly expand as 16S rRNA genes are sequenced from environmental samples and comparisons to existing data are drawn. Culture-independent methods have enabled the application of a molecular-phylogenetic approach to discover that the environmental microbes constitute the majority of evolutionary diversity [1]. Phylogenetic studies have further recognized that over 99% of all prokaryotes are uncultivated [2,3]. Therefore, in attempts to continue to explore the evolution and composition of microbial communities it is now standard practice to sequence the 16S rRNA gene [4].

These DNA sequences are being added to public databases rapidly since they have become the most cost effective, if not the only method available to identify and quantify the uncultivated microbes. Deriving meaningful knowledge from the inundation of sequences, then, necessitates methods to judge which sequences are of particular interest to the study. A common method in screening DNA sequences derived in a study is to assign each sequence to a taxonomic group by comparing it to the closest relative in publically available databases, such as Greengene’s Simrank [5] and NCBI’s BLASTN [6]. Although this method is rapid, it has been recognized that neither the top ranking hits nor the most similar sequences are always the most closely related phylotype [7,8]. 

Phylotypes may also be determined to be closely related by examining the network of sequence relationships based on their similarities [9]. A network is established using the following principle: if sequences *A*, *B*, and *C* each return sequence *a* as a BLASTN hit (with varying ranks), sequence *a* is considered to be “associated” with the three sequences. In the context of screening the sequences derived from environmental samples, analyzing the associations between the BLASTN hits of a collection will unveil sequences of great phylogenetic interest despite the possibility of having scored low in the BLASTN ranking system [10]. Recognizing these associations before aligning large collections of sequences reduces both the computational expense and the obfuscation that many sequences will inherently contribute to its analysis.

Here we introduce a web application, *Phylometrics*, which automates many key tasks for DNA sequence analysis in a high-throughput method. Instead of single BLASTN inquiries, Phylometrics automatically analyzes multiple BLASTN results, cross-comparing each BLASTN result against others within seconds. A graphical tool for mining these sequence relationships is also provided and includes them in a multiple sequence alignment process according to user-defined parameters. This approach can discover sequences of great interest for further phylogenetic examination which would otherwise be missed without significant human involvement. Finally, the application generates phylogenetic trees according to numerous methods of tree inference which may be selected and configured using a wizard-style form. As the results are being generated, the user may continue to interact and queue additional analyses without interrupting the ongoing processes.

Phylometrics offers a number of key advantages: simplicity in the user interface, powerful analytics to examine sequence relationships, the ability to install the application locally and the ability to queue batch jobs. The code is also released as open source allowing it to be extended and integrated into workflows as desired.  Finally, due to the architectural decision to make the application driven by job queues (a list of tasks to be run asynchronously), cloud computing services may be integrated and directed by the queue to perform the more CPU-intensive processes such as multiple sequence alignments.

## Implementation

### Design aims

Many popular tools available for sequence analysis (such as PHYLIP [11] and PhyML [12]) require command-line syntax and cryptic parameter settings that are foreign to biologists without programming experience. We identified four design aims for the system:

1. Implement a simple, widely usable interface that does not involve user programming, by implementing a wizard-styled approach.

2. Provide rich, interactive visual representations of the data generated by the pipeline.

3. Allow for the import of sequences in multiple formats and the export of data at every stage of the pipeline so as to give the user the flexibility to use alternative software solutions for those steps. Furthermore, the system must maintain a record of the parameters the user set for each step in the pipeline.

4. Allow batch jobs to be queued and/or running while being able to simultaneously create new jobs or able to access completed reports.

**Figure 1:**
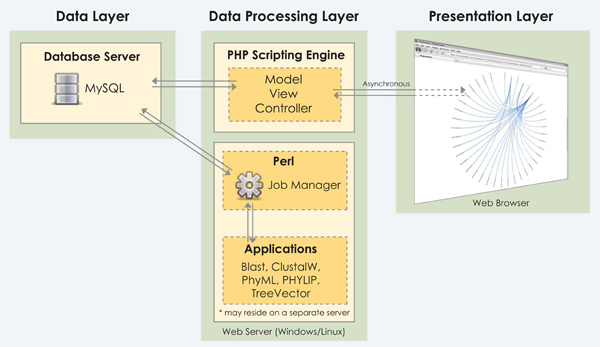
**Architectural overview of Phylometrics.** There are three main components to the system which are fully decoupled enabling them to be run on different hardware: the data, processing and presentation layers. The data processing layer may also be split in order to run high-computational applications on a different server.

These goals were met by architecting Phylometrics with three main components: the web browser, a script-processing engine running on a web server and a database server (see Figure 1). A Flash-enabled web browser, without any other plugin requirements, acts as the user interface and offers the unique advantage of not requiring a high-performance device and also allows the system to be accessed remotely. The back end is coded in PHP using a Model-View-Controller object-oriented programming paradigm, making it an extendable application. Phylometrics uses the open source MySQL database server to store sequence collections and reports. 

### User interface and pipeline wizard

A common web browser serves as the user interface. It is advantageous due to the low learning curve and accessibility from a number of devices. It also incorporates simple yet useful features such as printing and searching from within the browser experience. 

**Figure 2:**
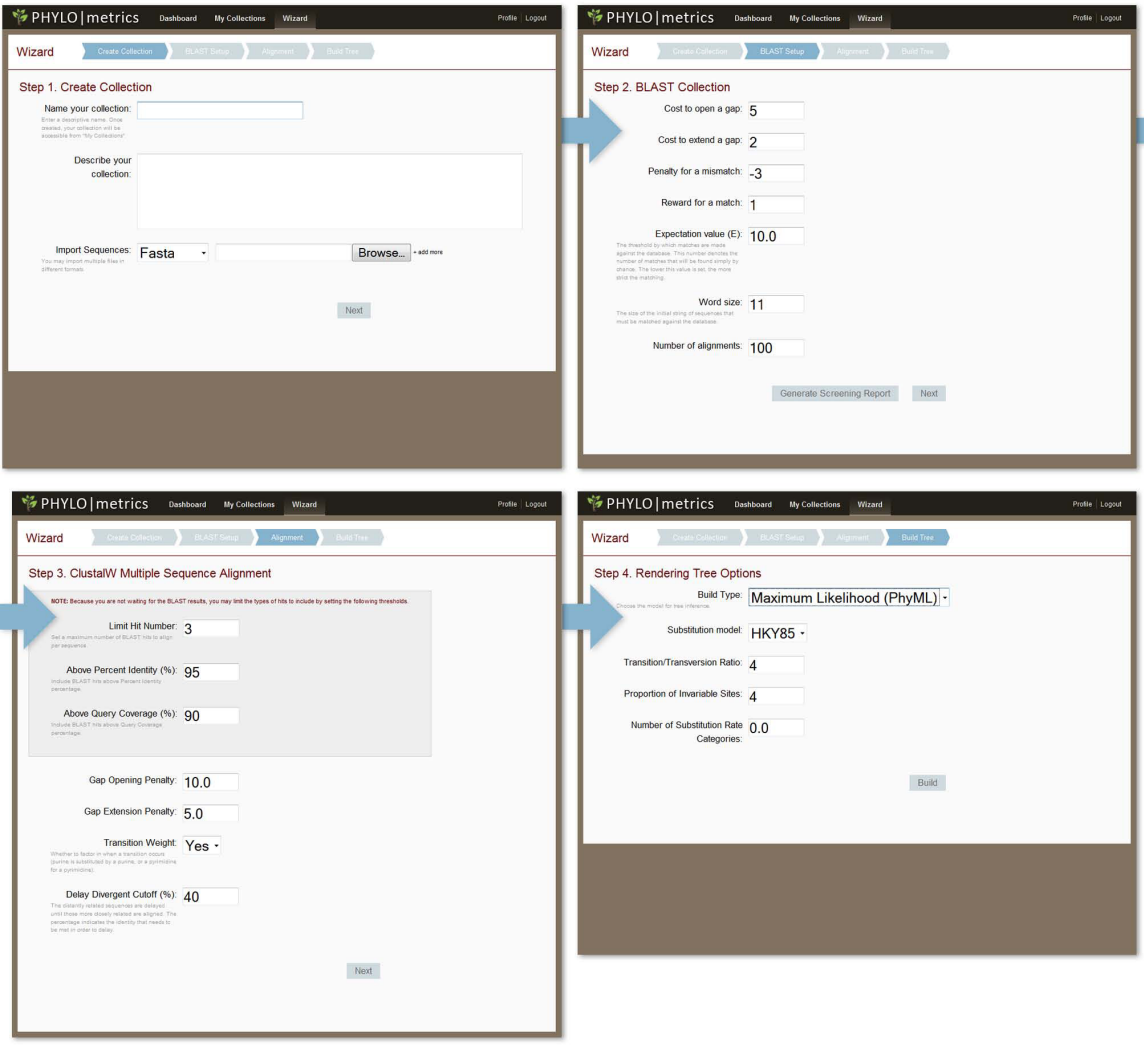
**Wizard-style data input.** The wizard process guides the user through four steps to explore phylogenetic relationships from a group of phylotypes: collection creation, BLAST setup, multiple sequence alignment parameters and tree inference algorithm selections. The interface provides visual contextualisation of the process, while allowing complex many-to-one relationships between collections, alignments and trees to be made simply.

Phylometrics implements a Wizard-style pipeline which prompts the user for parameters necessitated at each pipeline stage (see Figure 2). The first step in the process is creating a new “Collection” by importing sequences for analysis. On importing, a sequence record is created for each sequence and associated with a Collection record which is then pushed through the pipeline.

The second step of the Wizard is “BLAST Setup.” The software queries NCBI’s live database by default; this may be modified, however, to search a local BLASTN database instead. After entering BLASTN parameter options, the user has the ability to “Generate a Screening Report,” or to continue with the pipeline. Both processes are queued and allow the user to continue to use the system, while the option to generate the screening report will halt the pipeline at this second step before continuing the progression through the pipeline. A Screening Report will be generated irrespective of the choice the user makes at this point, allowing one to revisit this report to create any number of alignments and trees based on their filters.

The following step is “Alignment” with the options to select parameters based on ClustalW [13], a multiple sequence alignment program. Once the Screening Report is generated, users may choose to create alignments using various filters which set thresholds, gate and also specifically toggle individual sequences. In fact, one can use the same dataset to create many alignments using different parameters and compare the results in the generated trees.

The final step of the Wizard is “Build Tree”, where one can choose between various methods of tree inferences based on the PHYLIP [11] and PhyML[12] software packages. The NEWICK file format outputs of these packages are then rendered into images using TreeVector [14]. Similar to the many-to-one relationship of alignments to collections, many trees can be generated from one alignment. 

### Interactive visual analytics

**Figure 3:**
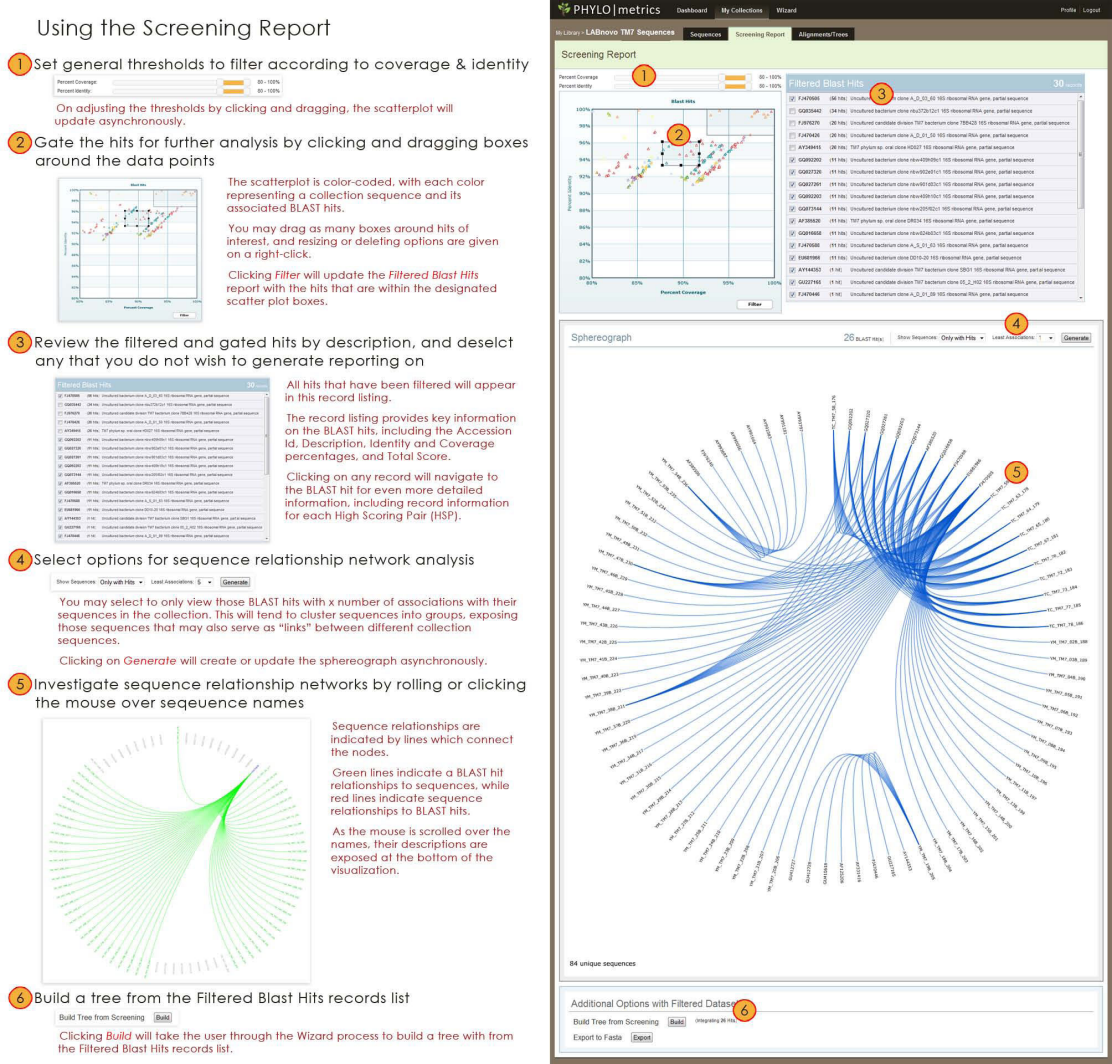
**Screening Report.** A screenshot of a typical Screening Report with an illustrated stepwise process for utilizing the report.

An interactive analytical tool developed in Adobe Flex and utilizing the Flare library is used to represent sequence relationship networks (see Figure 3). The user is prompted to select a number of filtering criteria from which a report is generated, including the minimum number of associations a BLASTN hit must have with individual supplied sequences. The report itself is interactive; scrolling over a sequence name with the mouse will reveal the sequence accession identification and description. Clicking a sequence name will highlight the relationship associations with other sequences based on the results obtained in BLASTN reports.

### Data usability

Sequences are initially imported by the user into a “Collection” from the Wizard in FASTA, Genbank or Raw format. On Wizard completion, the user is be able to download the BLASTN report in XML format, the multiple sequence alignments in PHYLIP format, and the tree inferences in NEWICK, SVG and PNG file formats. The user may also click on the generated results to review the parameters that were used to generate the reports within the Wizard process.

### Asynchronous job queues

The pipeline processing and job queues are managed by Perl scripts which utilize the BioPerl library [15]. These scripts are decoupled from the system in such a way so as to allow them to be run on a completely separate hardware platform, receiving job instructions from the database layer. This setup allows the user to continue to access and analyze generated reports while jobs are processing, in addition to being able to queue an unlimited number of jobs. As the tasks progress through the pipeline an unobtrusive panel displays the progress of each submitted job. Furthermore, this setup can be exploited to process jobs which are computationally expensive on other servers simultaneously or even utilize cloud computing services such as Amazon EC2.

## Results and discussion

We have compared the features of Phylometrics to a number of tools that are commonly used for small-subunit RNA analyses that offer differing advantages (see Table 1). Many phylogenetic tools (such as MEGA [17] and STAP [18]) utilize standard programs such as ClustalW and PhyML to perform common tasks. Phylometrics utilizes these and other standard packages (such as PHYLIP), and therefore does not differ from other pipelines or packages in these core functions. Thus, the performance on identical hardware will be similar. For instance, a representative collection of 20 sequences pushed through the full pipeline with default settings required 5.5 minutes on a Windows Server with a modest 2.66 Ghz single-core processor and 1GB RAM. Phylometrics differentiates itself from these tools by providing a novel method for discovering sequences that are ranked low yet may be phylogenetically significant in a high-throughput manner. 

**Table 1:** Comparison of Phylometrics to existing commonly-used tools for generating phylogenetic trees.

	Phylometrics	STAP	ARB	MEGA 4.1
User Interface	Web Browser	Command line	GUI	GUI
Where is it installed?	Local or Remote	Local	Local	Local
Open source	Yes	Yes	No	No
Offers Manual Alignment	No	No	Yes	Yes
Parallel Processing	Yes	Yes	No	No
Batch Jobs	Yes	Yes	No	No
Visualization of Sequence Networks	Yes	No	No	No

Phylometrics makes it possible to explore sequence relationships within a collection in order to quickly build phylogenetically meaningful trees. Using a visual and interactive interface, sequences that may provide key insights into functional or evolutionary relatedness are identified much faster than the current standard manual approach. Furthermore, the identification of homologous sequences previous to multiple sequence alignments and tree building significantly reduces computational costs and time by minimizing the inclusion of unrelated sequences.

Once a dataset has been refined and pertinent sequences have been identified, Phylometrics enables one to explore the differences between alignments and each method of tree inference to determine which set of methods and parameters may suit the data best. The parameters used in each of the processes are also permanently stored, allowing one to replicate the results manually. 

Results can be exported from any stage in the pipeline for personal collection databases or to be further analyzed in other popular sequence analysis packages, such as ARB [16]. The formats of the output include: Phylip for alignments, Newick for tree inferences and JPG and SVG formats for tree visualizations. The multiple sequence alignments are especially useful for export, as manually aligning sequences may be necessary to improve the quality of the resulting tree. The visual representations are also printable from within the Flash movie by accessing the menu with a right-click.

## Conclusions

Phylometrics offers an automated, high-throughput pipeline for phylogenetic inferences in a simple wizard-style web interface, requiring basic computer skills and no user programming experience. It incorporates novel analytical tools to mine sequence collections for biologically significant associations generating trees that provide insights into their respective functional or evolutionary implications.

## Availability and requirements

Project name: Phylometrics

Project home page: http://www.phylometrics.com

Operating system: Platform independent

Programming Language: PHP, Perl, Javascript

License: GPL

## Authors' contributions

SAS developed the application, and drafted the manuscript. CCO contributed to the conception of the project and manuscript.

## Competing interests

The authors declare that they have no competing interests.
